# The Abundance of Human Milk Oligosaccharide (HMO)-Metabolizing Genes in Fecal Samples from Six-Month-Old Human Infants

**DOI:** 10.3390/microorganisms9071352

**Published:** 2021-06-22

**Authors:** Lynn E. Ferro, Kameron Y. Sugino, Vanja Klepac-Ceraj, Sarah S. Comstock

**Affiliations:** 1 Department of Food Science and Human Nutrition, Michigan State University, East Lansing, MI 48824, USA; ferroly1@msu.edu (L.E.F.); suginoka@msu.edu (K.Y.S.); 2 Department of Biological Sciences, Wellesley College, Wellesley, MA 02481, USA; vklepacc@wellesley.edu

**Keywords:** human milk oligosaccharides, *Actinobacteria*, *Bifidobacterium infantis*, breastfeeding, human milk, infants, microbiota

## Abstract

Herein, we report the abundance and prevalence of HMO-metabolizing genes, specifically those of *Bifidobacterium infantis*, in fecal samples from human infants. Forty dyads were enrolled, and each mother collected a fecal sample from her infant at six months of age. Genomic DNA was extracted, and quantitative real-time PCR was used to determine gene abundance. The mode of delivery was not associated with gene abundance. Several gene regions, Sia (a sialidase), B. inf (16S), and GH750 (a glycoside hydrolase), were more abundant in the feces of human milk-fed infants (*p* < 0.05). Others, Sia and HC bin (16S), tended to be less abundant when a larger percentage of an infant’s diet consisted of solids (*p* < 0.10). When accounting for solid food intake, human milk exposure was positively associated with Sia and B. inf (*p* < 0.05) and tended to be related to the abundance of the GH750 and HC bin (*p* < 0.10) gene regions. With further development and validation in additional populations of infants, these assays could be used to group samples by dietary exposure even where no record of dietary intake exists. Thus, these assays would provide a method by which infant human milk intake can be assessed quickly in any well-equipped molecular biology laboratory.

## 1. Introduction

Mother’s milk contains the optimal nutrients for the growth and development of neonates. However, some infants are fed formula rather than human milk. Infant formula is an alternative option produced as a substitute for human milk. Generally, formulas are made from cow’s milk or soy milk [1]. These formulas are engineered to be similar to human milk in terms of macronutrient and micronutrient content and efforts to make formulas as similar to human milk as possible are underway [2]. However, many components found in human milk such as human milk oligosaccharides (HMOs) are still missing from most modern infant formulas [3,4,5,6]. For the most part, HMOs are not digested by infants but act as substrates for specific microbes, thereby shaping the overall composition of the infant’s gastrointestinal microbiome. Additionally, HMOs benefit infants consuming human milk due to their ability to block specific pathogens from attaching to the surface sugars of epithelial cells [7]. This can prevent disease in the gastrointestinal tract and even protect infants from respiratory and urinary tract infections [8].

Along with the functions of HMOs listed previously, HMOs support the growth of gastrointestinal microorganisms, primarily those from the genus *Bifidobacterium.* The gastrointestinal tracts of infants born at term and fed human milk are colonized mostly by *B. longum*, *B. longum* subspecies *infantis* (*B. infantis)*, and *B. breve* with small amounts of *B. bifidum* and *B. pseudocatenulatum* [9,10,11]. However, infants born at term that are formula-fed have a more diverse *Bifidobacterium* population. These diverse populations include *B. longum*, *B. breve*, and *Bifidobacteria* species more commonly seen in adults, such as *B. adolescentis* [9].

Of all infant gut microbes, *B.* *infantis* is one of the most comprehensively studied due to its ability to use HMOs as its sole carbon source [12]. As mentioned, *B. infantis* is commonly detected in breastfed infants. As such, *B. infantis* contains a large gene cluster and loci that are devoted specifically to the digestion of HMOs [12]. Most *Bifidobacterium* metabolize lacto-*N*-tetraose (LNT), a tetrasaccharide that is a core structure found in HMOs with a higher molecular weight; *B. infantis* also uses lacto-N-neotetraose (LNnT) [13]. LNT is typically present in a very high concentration in human milk during lactation [14]. *B. infantis* is able to consume LNT completely; however, *B. longum*, *B. breve*, and *B. bifidum* degrade LNT to a lesser extent. Most adult-associated strains of *Bifidobacterium* such as *B. adolescentis* and *B. animalis* do not degrade LNT or any other HMO compounds [12].

The main objective of this study was to determine if the HMO-metabolizing gene abundances in the gastrointestinal microbiota of six-month-old human infants are associated with early-life exposures such as type of feeding and mode of delivery. First, we hypothesized that infants who test positive for *B. infantis* HMO-metabolizing genes would have consumed a larger percentage of human milk in their diet. Secondly, if the vagina is a source of *B. infantis*, infants delivered vaginally would be more likely to have high levels of *B. infantis* HMO-metabolizing genes. These hypotheses were tested by screening fecal samples collected from infants for HMO-metabolizing genes by extracting genomic DNA and using quantitative real-time PCR to measure the prevalence and abundance of specific genes in the genomic DNA.

## 2. Materials and Methods

### 2.1. Study Design and Setting

This study followed a cross-sectional design but was nested within a prospective longitudinal pregnancy and birth cohort. This study serves as a secondary analysis and was conducted using a subset of participants described by Sugino et al. in 2020 [15]. The participants in this study resided in areas within and surrounding Lansing and Traverse City, Michigan, USA.

### 2.2. Participants

#### 2.2.1. Study Population and Sample Collection

Subjects: The women enrolled in this study were part of the ARCH GUT (*n* = 24) or BABY GUT (*n* = 16) cohorts. ARCH GUT is a part of the ARCH (Archive for Research in Child Health) study. The ARCH cohort recruited its participants from one clinic in Lansing and one clinic in Traverse City. The BABY GUT study recruited participants from several clinics in the greater Lansing area. Study procedures for ARCH (IRB #C07-1201), ARCH GUT (IRB #14-170M), and BABY GUT (IRB #15-1240) were approved by the Michigan State University’s IRB.

Sample collection: Collection kits were assembled at the research facility and sent to the participants by mail. The collection kits included instructions for taking a fecal sample, a ParaPak tube for sample collection (Meridian Bioscience, 900312, Cincinnati, OH, USA), or diapers for the infant sample. The mothers collected fecal samples from the infant’s diaper at home. The infants were near six months of age at the time of sample collection. Forty samples were included in the final analysis. The samples were sent to the laboratory by mail or retrieved from the home, and fecal aliquots were stored at −80 °C upon reaching the laboratory.

#### 2.2.2. Diet Analysis

In a survey, the participants estimated the fraction of their infant’s diet over the past week that was human breast milk as 100%, 80%, 50–80%, 50%, 20–50%, 20%, or 0% [16]. Due to the low number of individuals, the infants were categorized as having any (*n* = 28) or no (*n* = 12) exposure to human milk. The mothers also recorded information regarding whether the infant ate solid foods and if so, what foods were consumed. Using this information, the infants were categorized into one of the three categories describing their solid food intake: no solids, few solids, many solids. The “few solids” category was defined as having some cereal and/or one fruit/vegetable in the diet while the “many solids” group was defined as a larger variety (more than two) of solid foods.

#### 2.2.3. Sample Storage and DNA Extraction

Genomic DNA from the fecal samples was extracted using a DNeasy PowerSoil Pro Kit from Qiagen (Carlsbad, CA, USA) as described by Sugino et al. [17]. After the extraction, the genomic DNA samples were stored at −80 °C until testing.

### 2.3. Quantitative Real-Time PCR

Each gDNA sample (*n* = 40) was screened for the presence of total 16S rRNA/DNA, the *Bifidobacterium* genus, *B. infantis*, and *B. breve*. All 40 samples were screened using 11 different primer sets. All the genes and their respective primer sets as well as functions are further described in Table 1. The development of these primer sets was described by Tso et al. [18]. DNA was isolated from commercial probiotics containing the bacterial strains of interest to optimize assay conditions for each set of primers. Once determined, those primer conditions were used for the remaining experiments. Each well, when run, contained 15 μL in total (Supplementary Materials Table S1). The volume of forward and reverse primers, amount of SYBR green, gDNA, and DNA-free water was dependent on the concentration necessary for each specific primer set. Supplementary Materials Table S1 outlines the reaction volumes per well and the optimal primer concentration (300 µM, 1 µM, 5 µM, 12.5 µM).

PCR thermocycler conditions for each primer set, Sia-266F676R, B. inf, g-Bif, GH-492F1002R, Inf2348, HC bin F, Uni, GH750F1258R, HH-60F534R, Blon0915, and Bbreve are described in Supplementary Materials Table S2. The annealing temperature is specific for each primer set and is listed in Table 1.

**Figure 1:**
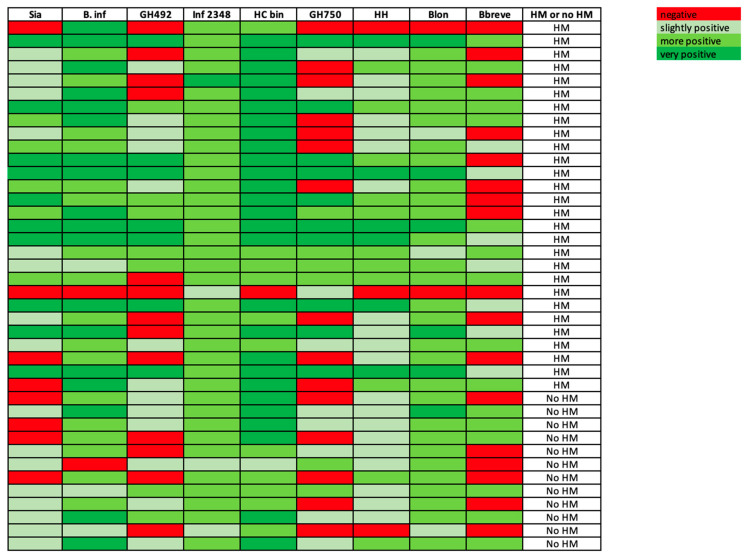
Heat map of the HMO-metabolizing genes detected in each of the infant stool samples. The colors show the range from negative (red) to very positive (dark green), with the top portion of the map being infants fed some amount of human milk, and the infants in the bottom portion not consuming any human milk at the time of sample collection.

Following kinetic fluorescent quantification using a QuantStudio 7 Flex qPCR instrument (Applied Biosystems; Foster City, CA, USA) in the Michigan State University’s Genomics Core, QuantStudio7 (Applied Biosystems) was used to identify and export C_T_ values. The lower the C_T_ value, the more abundant the gene. The ranges of negative to very positive were calculated via the C_T_ value, with the following cutoffs: negative, >32; slightly positive, ≥28 and <32; more positive, ≥20 and <28; very positive, <20 (Figure 1).

### 2.4. Statistical Analyses

Basic descriptive statistics and proc GLM to compare gene abundance by human milk, solid food intake, and mode of delivery were conducted using SAS (Cary, NC, USA; version 9.4); *p* < 0.05 was considered significant; *p* < 0.10 was considered a trend. Rarified count data of individual taxa with greater than 1% average relative abundance were compared using a negative binomial model in the MASS package (Venables and Ripley, 2002). The Mann–Whitney test was used to compare the number of HMO genes for each infant categorized as having any or no human milk intake.

## 3. Results

### 3.1. Participant Characteristics

Forty women (*n* = 40) participated in this study and submitted infant stool samples (Table 2). The mean age of the women was 31.79 ± 4.54 years, and the mean age of the infants was 202.53 ± 22.56 days. Of the infants, 62.5% (*n* = 25) were delivered vaginally, and 70% (*n* = 28) were male. For human milk exposure, 70% (*n* = 28) of the infants were exposed to some human milk and 30% (*n* = 12) had not been exposed to any human milk in the week immediately prior to stool sample collection. Infant solid food intake was also reported by the women participating in the study. Eighty-five percent (*n* = 34) of the infants were reported to have consumed some type of solid food. The mothers also reported the solid foods that the infants ate. The most commonly reported were vegetables (60%), fruit (57.5%), rice (27.5%), oats (20%), and meat (5%) (Table 2).

### 3.2. Gene Prevalence and Abundance

When analyzing gene prevalence and abundance in infants who consumed any amount of human milk versus no human milk, Sia was more prevalent and abundant in those infants who consumed human milk (Figure 1). This relationship was also true for B. inf (Figure 1). Of those consuming human milk, 85.7% (*n* = 24) were positive for Sia, while 96.4% (*n* = 27) were positive for B. inf. Overall, 80% (*n* = 32) of the infants were positive for Sia, but in the samples from infants fed no human milk, Sia was either undetectable or slightly positive. Of the infants whose samples contained detectable levels of B. inf (95% (*n* = 38)), 25% of those from the infants fed no human milk were very positive for B. inf whereas 54% of those from the infants fed human milk were very positive. Inf 2348 was detected in all the infant samples (*n* = 40) while HC bin was detected in all but one sample (*n* = 39). HH (haloacid dehalogenase-like hydrolase domain-containing protein) was detected in 92.5% (*n* = 37) of the infants, Blon (major facilitator superfamily)—in 95% (*n* = 38) of the infants. GH492 (glycoside hydrolase), GH750 (glycoside hydrolase), and Bbreve (16S) were detected in fewer infants—67.5% (*n* = 27), 62.5% (*n* = 25), and 57.5% (*n* = 23) respectively. When we considered detection of the following five genes, Sia, GH492, Inf 2348, GH750, and HH, at a level that was “more positive” or “very positive,” we found that the stool of the human milk-fed infants harbored a more diverse set of HMO genes than that of the non-human milk-fed infants (*p* = 0.0148). The stool samples from the human milk-fed infants contained a median of 2.5 of the five HMO genes whereas the samples from the non-human milk-fed infants contained a median of one HMO gene.

### 3.3. Gastrointestinal Bacterial Communities

Supplementary Materials Figures S1–S4 display bar charts of the gastrointestinal bacterial communities at the genus and phylum levels separated by the extent of solid food and human milk consumption. Infants consuming any amount of human milk tended to have a larger abundance of Actinobacteria (phylum) (*p* = 0.088; Supplementary Materials Figure S3) and *Bifidobacterium* (genus) (*p* = 0.087; Supplementary Materials Figure S4) in their gastrointestinal microbiome. When comparing taxa according to the extent to which infants ate solids (no solids, little solids, or some solids), Firmicutes were enriched in the solid food eaters (*p* = 0.011; Supplementary Materials Figure S1). However, there were no significant differences in *Bifidobacterium* (genus) or Actinobacteria (phylum) abundances in accord with the extent of solid food consumption (Supplementary Materials Figures S1 and S2).

### 3.4. Univariate Models

**Figure 2:**
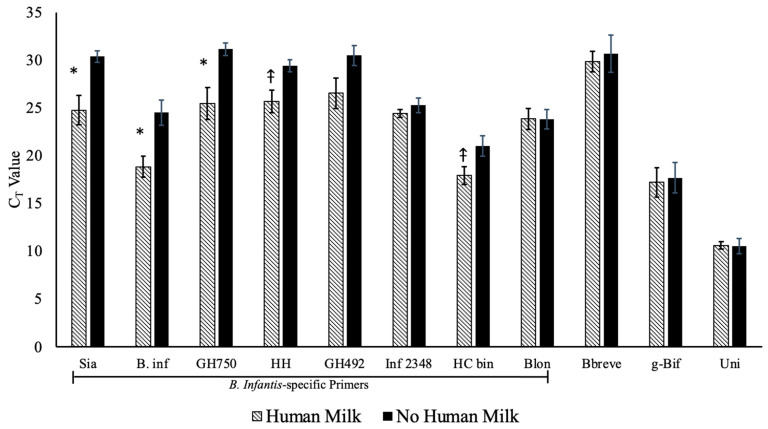
The abundance of each gene (displayed as the Ct value) for the infants who consumed any amount of human milk versus those who consumed no human milk. The data are means ± SD; * *p* < 0.05; ⤉ *p* < 0.10. Note that the higher the C_T_ value, the lower the gene abundance.

We first analyzed the relationship between the HMO-metabolizing genes and the mode of delivery, human milk exposure, or solid food consumption in univariate models. No genes were significantly associated with the mode of delivery. However, the bacterial DNA isolated from the stool of the breastfed infants had a greater abundance of the Sia (*p* = 0.0235), B. inf (*p* = 0.0047), and GH750 (*p* = 0.0372) genes compared to the non-breastfed infants (Figure 2). The abundance of the genes HC bin (*p* = 0.0597) and HH (*p* = 0.0523) also tended to be positively associated with breast milk consumption (Figure 2). When measuring the relationship between the abundance of each gene and the extent to which the infants ate solid foods, no gene abundances were associated with the extent of solids consumed.

### 3.5. Multivariate Model

**Figure 3:**
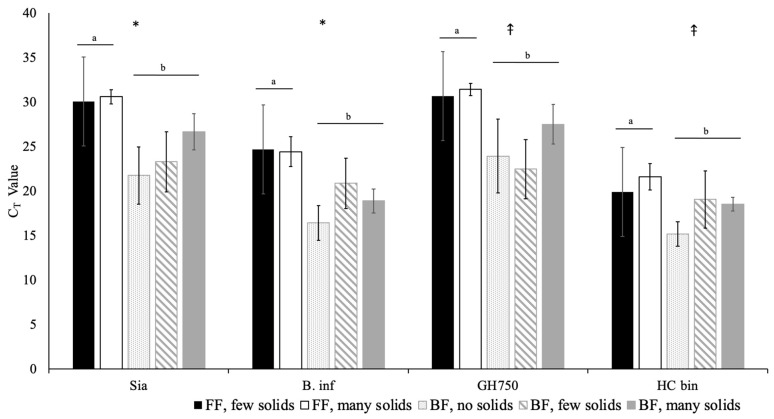
The abundance of each gene (displayed as the C_T_ value) whether the infants were formula-fed (FF) or breastfed (BF) and the amount of solids that the infants consumed (no solids, few solids, many solids). Solid food intake was not significantly associated with the abundance of any gene. For Sia and B. inf, one is still able to determine if the infants consumed human milk as part of their diets, even once they start eating solid foods. Note that the higher the C_T_ value, the lower the gene abundance. The data are means ± SD; * *p* < 0.05; ⤉ *p* < 0.10. Within a gene, the bars that do not share a letter differ significantly from each other.

Because the combination of breast milk and solid food consumption represents the full extent of the infant diet, these variables were then considered in a multivariate model (Figure 3). When accounting for solid food intake, human milk exposure continued to be associated with Sia (*p* = 0.0247) and B. inf (*p* = 0.0165) gene abundance. Human milk exposure also tended to be associated with GH750 and HC bin gene abundance when accounting for solid food intake (Figure 3). The interaction between the consumption of human milk and the extent to which the infant ate solids was not significant, and therefore the interaction was not included in our final models.

## 4. Discussion

In this study, we determined the abundance and prevalence of HMO-metabolizing genes in the gastrointestinal microbiota of infants at six months of age. The genes found in *Bifidobacterium infantis* were of special interest in this study due to the ability of these bacteria to digest HMOs. Exposure to human milk was positively associated with the abundance of the genes Sia and B. inf and tended to be positively associated with GH750 and HC bin gene abundance as well. Collectively, these data demonstrate that the genes Sia or B. inf are strong markers of HMO intake and therefore of human milk intake. Sia may be the more discriminatory marker. However, further development and validation of the assays is necessary.

Herein, the mode of delivery and the abundance of the HMO-metabolizing genes were not significantly related. However, other research studies that assessed this relationship reported different results. For instance, the abundance of *Bifidobacterium* (as determined by 16S rRNA gene sequencing) was significantly lower in the infants delivered by Cesarean section compared to the vaginally delivered infants at ages of 10 days [25], three weeks [26,27], and one month [28]. While these results differ from what was observed in our study, this is likely due to the age of the infants at the time of sampling. Our study analyzed *Bifidobacterium* genes in samples taken from infants at six months of age, a much later age period compared to other studies. Our results are similar to reports from infants three months and older, which also show no significant difference in *Bifidobacterium* abundance by the mode of delivery [29,30]. Differences were observed in the relative abundance of *Bacteroides* at birth related to the mode of delivery; however, as the infants aged, the differences decreased, supporting the idea that any microbial difference from the mode of delivery has no long-term impact at six months of age [31,32,33,34]. Therefore, it is likely that we did not detect a difference related to the mode of delivery because our infants were six months old. Therefore, in order to determine whether the mode of delivery is associated with specific HMO-metabolizing genes, the samples collected from infants younger than six months of age need to be studied.

Our choice to study infants at six months of age was for a variety of reasons. As mentioned in the paragraph above, at the six-month timepoint, changes in the microbiome of infants due to the mode of delivery are unlikely to be detected; therefore, this variable could be removed from our multivariate analyses [29,30,31]. Additionally, due to a large variation in the amount of human milk infants consume at this age, we expected to detect variations in the abundance of HMO genes due to the amount of human milk consumed by the infants. However, due to the sample size and sensitivity of the assay, we did not have the power to detect these differences. A study with a larger sample size may be able to assess potential differences in HMO gene abundance by the proportion of human milk consumed in the diet. As long as the microbiome remains capable of being manipulated by prebiotics, probiotics, and diet at six months of age, interventions at this point in an infant’s life can lead to long-term modifications that do not require consistent administration of the treatment [31]. Finally, the number of infants fed human milk as well as the amount of human milk each infant is fed drops off as infants age. Due to the variability in the delivery method, feeding habits, and milk consumption, we chose six months of age as the timepoint for this study.

These results demonstrate that stool genomic DNA isolated from breastfed infants had a greater abundance of the Sia, B. inf, and GH750 genes (Figure 1) and a greater diversity of HMO genes Sia, GH492, Inf 2348, GH750, and HH. Other studies have shown that within the infant gastrointestinal microbiome, there are multiple species and strains of *Bifidobacterium* that exist together [35]. It has been reported that the consumption of HMOs is associated with a microbiota rich in *Bifidobacterium*, and that HMO consumption by bifidobacteria also affects the infant gastrointestinal microbiome [36]. In a study looking at HMO profile variation in healthy women postpartum where 89.1% of women were identified as secretors, women with a functional FUT2 gene, and 10.9% were non-secretors, it was found that the most abundant HMO in the milk of secretor women was 2′FL, and the total HMO concentration at 88–119 days postpartum was significantly higher than at 2–8 days postpartum [37]. Our study could be extended further by knowing the secretor status as well as the HMO profile of the participating mothers. This would allow us to elucidate the impact of HMOs on infant outcomes related to the variety of HMOs produced by the mother.

In our study, genes Sia or B. inf were markers of human milk consumption by infants or, more specifically, of HMO intake. However, there are many types of HMOs with varying composition in the human milk of women throughout the duration of lactation [37]. Analyses of the HMO variation in human milk up to three and four months postpartum demonstrate that HMO concentrations (g/L) decrease over time postpartum [37,38,39]. There is a relative decrease in high-molecular HMOs over time and an increase in low-molecular HMOs over time, therefore decreasing the overall HMO concentration in g/L over time postpartum [37,38,39]. Not only does the abundance and composition of HMOs differ across lactation stages for women, but there is also variation in the same woman over time [40]. Therefore, the composition of the milk the mother is producing and feeding to her child as well as the amount of time postpartum can impact the presence of HMO-metabolizing genes in the stool of the infant. As a result, this means any genes that encode HMO-metabolizing proteins specific for the HMOs that are not common in all human milk would be poor markers of human milk intake. On the other hand, the genes encoding HMO-metabolizing proteins specific for HMOs that are common in all human milk would be good markers of human milk intake. Thus, Sia encodes a sialidase that must attack a structure common to all human milk. However, the B. inf gene is not specific for an HMO-metabolizing enzyme. Rather, that gene encodes a specific sequence in the rRNA/DNA gene for a specific strain of *B. infantis*.

When one’s diet is not recorded, aspects of it can be determined using biomarkers, a common yet potentially expensive method. There is no single biomarker to determine an infant’s diet. In fact, many methods can be used to determine an infant’s diet [41]. Specifically, NMR-based metabolomics and other untargeted metabolomic methods have been used to investigate metabolites in biospecimens from infants fed either human milk, cow’s milk formulas, or soy-based formulas [42,43]. Thus, we would expect that the fecal metabolomes would be significantly different based on the diet; however, given that these infants were six months old at the time of sampling, it was not predicted that the mode of delivery would have a significant impact [43]. In our case, quantitative real-time PCR was used to assess the presence of the genes related to the infant diet composition. If the circumstances prevented us from collecting nutritional information from the mothers of the infants studied, it would still be possible to determine their breastfeeding habits and human milk consumption by analyzing the abundance of the genes Sia and B. inf. However, further development of these assays in a larger and more diverse population is required to confirm that the results reported herein are generalizable. The quantitative real-time PCR method used in this study is cheaper, faster, and simpler compared to metabolomics, shotgun metagenomic sequencing, or fluorescence in situ hybridization (FISH). Our method differs from other omics techniques in that it does not require knowledge of bioinformatics pipelines for the analysis of complex datasets, yet it provides a method of estimating the relative abundances of specific taxa [44]. If the correct primers are used, such as those used to detect Sia and B. inf in this study, this PCR-based method allows for the differentiation between human milk- and non-human milk-fed infants in a way that can be adequately and economically achieved. Other advantages to using PCR are that the test is fast, sensitive, specific (due to primer specificity), and provides detection and quantification of the DNA. Furthermore, cross-contamination is unlikely because the samples are not manipulated after they are amplified. These advantages make PCR a reliable alternative to the standard culture methods [45]. However, there are some limitations when studying bacterial genes using PCR. There is the possibility that any one bacterium could have multiple copies of each of the genes detected, and each bacterium could have a different number of each of these genes. Furthermore, the presence of genes does not provide information about their expression or production. PCR cannot distinguish between live and dead bacteria, preventing us from determining if the DNA that is detected is from the bacteria making a metabolic contribution in the gastrointestinal tract. Additionally, while PCR-based tests are fast, the results should be analyzed and correlated with phenotypic and biochemical tests [42]. These issues make it difficult to conduct a quantitative analysis of the actual metabolizing capacity for the HMOs, demonstrating possible pitfalls to using quantitative real-time PCR. While our study uses quantitative real time-PCR, other methods for the detection of *Bifidobacterium* in the intestine of infants include fluorescence in situ hybridization (FISH) as it is able to detect minute amounts of *Bifidobacterium*; however it requires specific equipment and expensive fluorescent labels and is therefore not as cost-effective as the method described herein [46,47].

### 4.1. Limitations

In addition to the potential issues associated with quantitative real-time PCR, other limitations of this study include a small sample size (*n* = 40) of women in a single geographic location, thereby limiting the generalizability of the results. As long as only 40 mothers and their infants were enrolled in this study, it was difficult to determine a threshold of abundance of these genes in relation to the extent of human milk the infants consumed.

### 4.2. Future Directions

Future studies will expand the number of infants as well as include infants of a variety of ages, such as one month and three months of age, to determine if HMO-metabolizing gene expression is sensitive to quantities of human milk as well as if factors such as the mode of delivery contribute to HMO-metabolizing gene expression and abundance. Other future directions could include determining the mother’s secretor status and HMO profile which would allow us to relate the impact of HMOs on infant outcomes to the variety of HMOs produced by the mother. Finally, future studies could determine if the mode of feeding results in fecal metabolite profiles that are unique to breastfed infants and whether those metabolites correlate with Sia gene abundance.

## 5. Conclusions

In conclusion, our study provides evidence that detection in infant stool of the gene targeted by the Sia primer set is indicative of human milk intake. Therefore, it is possible that banked stool samples could be used to determine infant diet composition in studies where diet data was not collected. More specifically, the PCR test could determine whether the infant consumed human milk at the time of stool sampling.

## Figures and Tables

**Table 1:** Primer Sets.

Primer Set	Forward and Reverse Primers (5′–3′)	Gene Targeted	Organism Targeted	Annealing Temperature (°C)	Primer Concentration	Reference
Sia	F: GACGAGGAGGAATACAGCAG R: CACGAACAGCGAATCATGGATT	Sialidase (Blon_2348)	*B. longum infantis*	58	1 μM	Klepac-Ceraj (unpublished)
B. inf	F: CCATCTCTGGGATCGTCGG R: TATCGGGGAGCAAGCGTGA	16S rRNA	*B. longum infantis*, *B. longum longum*, *B. indicum*	57	300 nM	Tannock et al. (2013) [19]
g-Bif	F: CTCCTGGAAACGGGTGG R: GGTGTTCTTCCCGATATCTACA	16S rRNA	*Bifidobacterium* genus	55	1 μM	Tannock et al. (2013) [19]
GH-492	F: CGATGATGTGCTGGATTCGTTC R: CTCGACCATTCCAAGATGCTA	Glycoside hydrolase (Blon_2358)	*B. longum infantis*	60	300 nM	Klepac-Ceraj (unpublished)
Inf 2348	F: ATACAGCAGAACCTTGGCCT R: GTTCTCGTCCATGTGATCGC	Sialidase	*B. longum infantis*	60	5 μM	Lawley et al. (2017) [20]
HC bin	F: AGGATACGTTCGGCGTC R: CGCAAGATTCCTCTAGCA	16S rRNA	*B. longum infantis*	60	5 μM	Hong and Chen (2007) [21]
Uni	F349: ACTCCTACGGGAGGCAGCAGT R528:ATTACCGCGGCTGCTGGC	16S rRNA	Targets all bacteria	60	1 μM	Tannock et al. (1999) [22]
GH750	F: GCGCCATCCTGGTGATGTTATT R: CTACGTGATCTGGGAGAGTTTC	Glycoside hydrolase (Blon_2355)	*B. longum infantis*	59	5 μM	Klepac-Ceraj (unpublished)
HH	F: CCACAATGTCATCGACCATCTG R: CCGAAGTATTCGGATGCCTATG	Haloacid dehalogenase-like hydrolase domain-containing protein (Blon_2356)	*B. longum infantis*	59	5 μM	Klepac-Ceraj (unpublished)
Blon	F: CGTATTGGCTTTGTACGCATTT R: ATCGTGCCGGTGAGATTTAC	Major facilitator superfamily	*B. longum infantis*	50	1 μM	Frese et al. (2017) [23]
Bbreve	F: CCGGATGTCCATCACAC R: ACAAAGTGCCTTGCTCCCT	16S rRNA	*Bifidobacterium breve*	55	5 μM	Matsuki et al. (1998) [24]

**Table 2:** Participant characteristics.

	Total *n* = 40
Maternal age, years	31.79 (min, 22; max, 39)
Vaginal birth, % (*n*)	62.5 (25)
Infant age, days	202.53 (min, 161; max, 292)
Male, % (*n*)	70 (28)
Human milk exposure, % (*n*)	
100%	17.5 (7)
20–80%	27.5 (11)
>0 but <20%	25 (10)
0%	30 (12)
Human milk, new	
Any HM, %	70 (28)
No HM, %	30 (12)
Solid food intake, % (*n*), yes	
No solids	15 (6)
Some solids	27.5 (11)
Significant solids	57.5 (23)
Solid food intake, food-specific, % (*n*), yes	
Rice	27.5 (11)
Fruit	57.5 (23)
Vegetables	60 (24)
Meat	5 (2)
Oats	20 (8)

## Data Availability

The data are not publicly available due to privacy concerns given the small sample size. However, the data presented in this study are available upon request from the corresponding author.

## Supplementary Materials

The following are available online at https://www.mdpi.com/article/10.3390/microorganisms9071352/s1, Table S1: Reaction volumes per well, Table S2: Conditions for qPCR, Figure S1: Phyla bar charts according to solid food intake, where 0 represents an infant consuming no solids, 1 represents an infant consuming little solids, and 2 represents an infant consuming some solids, Figure S2: Genera bar charts according to solid food intake, where 0 represents an infant consuming no solids, 1 represents an infant consuming little solids, and 2 represents an infant consuming some solids, Figure S3: Infants receiving any human milk tended to have a higher abundance of Actinobacteria than those receiving no human milk at all (*p* = 0.088), Figure S4: Infants receiving any human milk tended to have a higher abundance of *Bifidobacterium* than those receiving no human milk at all (*p* = 0.087).
