# Supplementary material for: The Abundance of Human Milk Oligosaccharide (HMO)-Metabolizing Genes in Fecal Samples from Six-Month-Old Human Infants
Source: Microorganisms. 2021 Jun 22;9(7):1352. doi: 10.3390/microorganisms9071352 (PMC8307860; doi:10.3390/microorganisms9071352)
Supplement: Supplementary file 1 [file microorganisms-09-01352-s001.zip › microorganisms-1240329-supplementary.pdf]

Supplementary Tables and Figures

Table S1: Reaction Volumes per Well

Component	Per well (tot vol 15µl per rxn)	Per well (tot vol 15µl per rxn)	Per well (tot vol 15µl per rxn)	Per well (tot vol 15µl per rxn)
**Primer concentration**	**300 nM**	**1 µM**	**5µM**	**12.5 µM**
SYBR green[1]	7.5 µl	7.5 µl	7.5 µl	7.5 µl
Forward Primer[2]	0.045 µl	0.155 µl	0.75 µl	1.9 µl
Reverse Primer	0.045 µl	0.155 µl	0.75 µl	1.9 µl
5ng Genomic DNA (samples)	2 µl	2 µl	2 µl	2 µl
DNA-Free Water[2]	5.4 µl	5.2 µl	4 µl	1.7 µl
**Total**	**15 µl**	**15 µl**	**15 µl**	**15 µl**

[1]Applied Biosystems (Foster City, CA); [2]IDT (Coralville, IA)

Table S2: Conditions for qPCR

	B. Breve		Remaining Primers	
Cycles	Temperature (C)	Time	Temperature (C)	Time
1x	94	30 sec	50	2 min
40x	94	30 sec	95	10 min
	55	1 min	95	45 sec
	68	30 sec	**X**[1]	45 sec
1x	68	5 min	72	45 sec
	4	HOLD	4	HOLD

[1]Where X is the primer-dependent annealing temperature

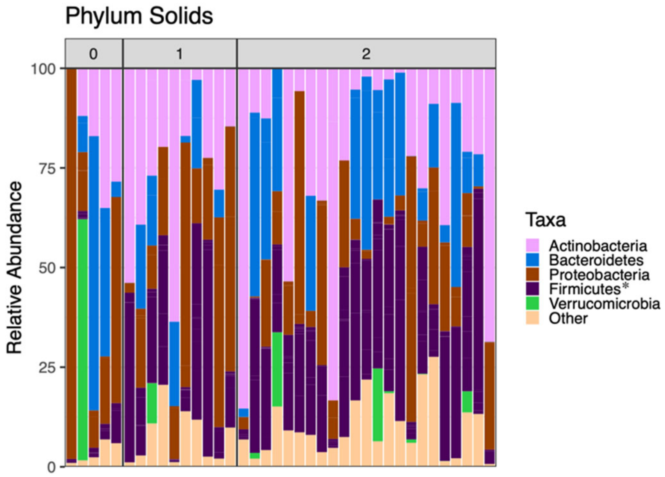

Figure S1: Phyla bar charts according to solid food intake, where 0 represents an infant consuming no solids, 1 represents and infant consuming little solids, and 2 represents and infant consuming some solids.

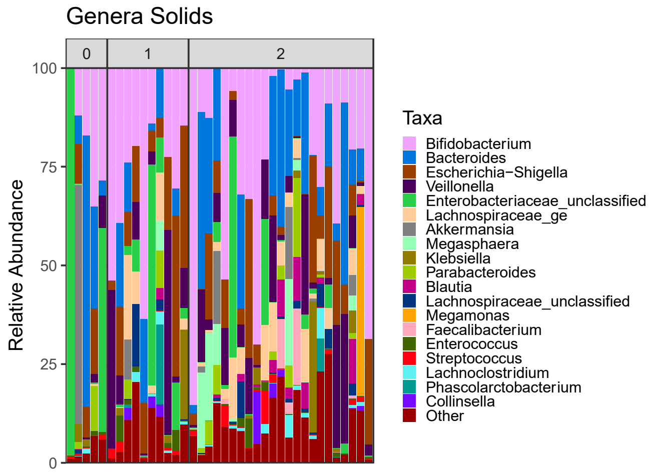

Figure S2: Genera bar charts according to solid food intake, where 0 represents an infant consuming no solids, 1 represents and infant consuming little solids, and 2 represents and infant consuming some solids.

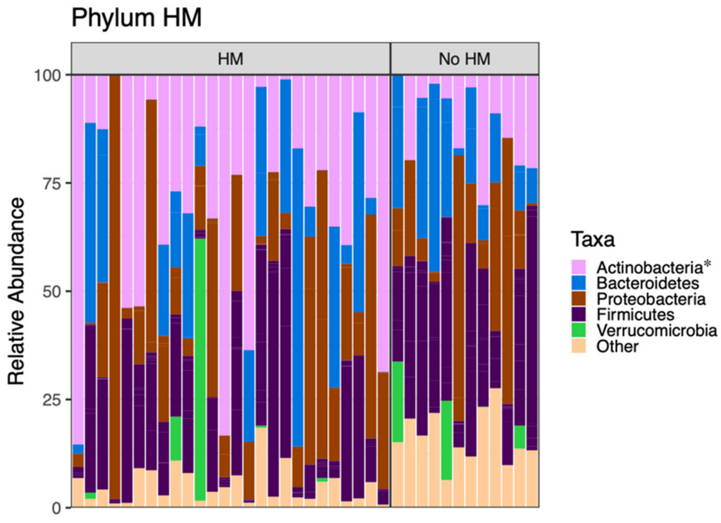

Figure S3: Phyla bar charts according to human milk intake. Infants receiving any human milk tended to have a higher abundance of Actinobacteria than those receiving no human milk at all (p=0.088).

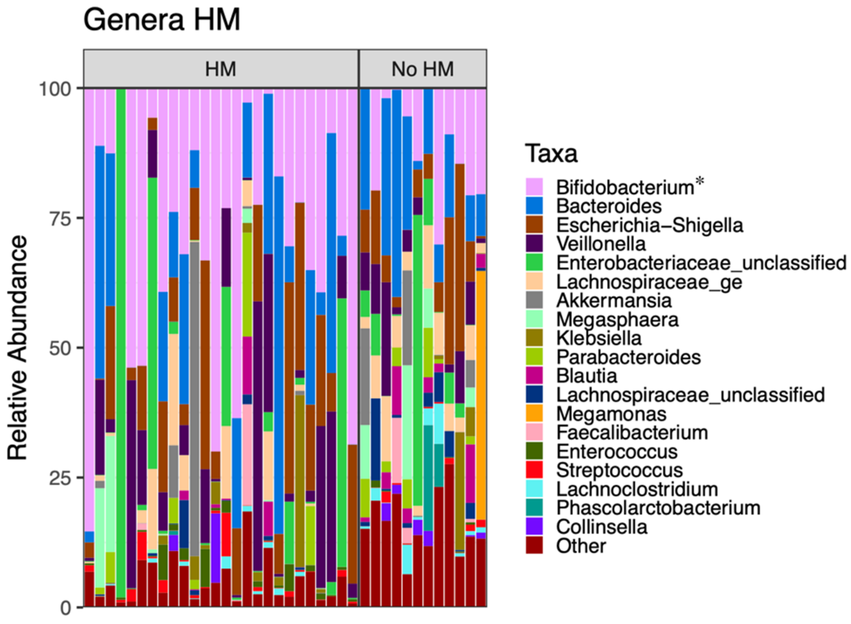

Figure S4: Genera bar charts according to human milk intake. Infants receiving any human milk tended to have a higher abundance of Bifidobacterium than those receiving no human milk at all (p=0.087).
